# When fat is not bad: the regulation of actin dynamics by phospholipid signaling molecules

**DOI:** 10.3389/fpls.2014.00005

**Published:** 2014-01-23

**Authors:** Roman Pleskot, Přemysl Pejchar, Christopher J. Staiger, Martin Potocký

**Affiliations:** ^1^Institute of Experimental Botany, v. v. i., Academy of Sciences of the Czech RepublicPrague, Czech Republic; ^2^Department of Biological Sciences, Purdue UniversityWest Lafayette, IN, USA

**Keywords:** actin, actin-binding proteins, capping protein, cytoskeleton, phosphatidic acid, phosphatidylinositol 4,5-bisphosphate, phospholipase D, signaling

## Abstract

The actin cytoskeleton plays a key role in the plant morphogenesis and is involved in polar cell growth, movement of subcellular organelles, cell division, and plant defense. Organization of actin cytoskeleton undergoes dynamic remodeling in response to internal developmental cues and diverse environmental signals. This dynamic behavior is regulated by numerous actin-binding proteins (ABPs) that integrate various signaling pathways. Production of the signaling lipids phosphatidylinositol 4,5-bisphosphate and phosphatidic acid affects the activity and subcellular distribution of several ABPs, and typically correlates with increased actin polymerization. Here we review current knowledge of the inter-regulatory dynamics between signaling phospholipids and the actin cytoskeleton in plant cells.

## INTRODUCTION

The plant actin cytoskeleton is a molecular scaffold that controls many aspects of cytoarchitecture including cytoplasmic streaming, movement and positioning of diverse organelles, or individual proteins. It also plays a prominent, albeit incompletely understood, role in endocytic and exocytic processes and has been implicated in cytokinesis, polar growth, and defense responses to pathogens (Higaki et al., 2007). Actin filaments are generated from monomeric actin subunits (G-actin) and arrayed into dynamic networks in plant cells; actin turnover and the formation of higher-order structures is tightly regulated by dozens of actin-binding proteins (ABPs). These proteins can be divided into several groups according to their binding properties and activities, e.g., monomeric G-ABPs; capping and severing proteins; side-binding proteins; and actin-nucleating factors (Staiger and Blanchoin, 2006; Henty-Ridilla et al., 2013).

To ensure proper spatial and temporal regulation of actin dynamics, the activity and binding properties of ABPs are further modulated by upstream-signaling molecules (reviewed, e.g., in Thomas et al., 2009; Blanchoin et al., 2010; Fu, 2010). Here we review the role of minor signaling membrane components, phosphatidylinositol 4,5-bisphosphate (PIP2), and phosphatidic acid (PA), that have been discovered as important regulators of actin dynamics in plant cells. In particular, we address the following subjects: (i) characteristics of PIP_2_ and PA that permit their function in cells; (ii) specific production of actin-regulating PIP_2_ and PA pools; (iii) current knowledge on the regulation of different ABPs mediated by direct interaction with PIP_2_ and/or PA; and (iv) putative crosstalk between PA and PIP_2_ in the regulation of actin dynamics.

## UNIQUE STRUCTURAL PROPERTIES OF PIP_2_ AND PA DETERMINE THEIR BIOLOGICAL ACTIVITY

**FIGURE 1:**
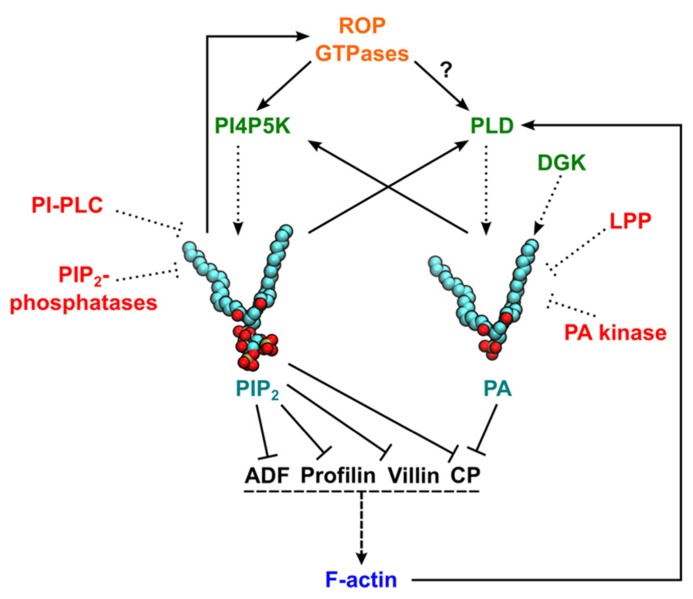
**Schematic representation of actin regulation by phosphatidylinositol 4,5-bisphosphate (PIP_2_) and phosphatidic acid (PA).** Solid lines represent pathways leading to activation (arrow ends) or inhibition (line ends). Dotted lines indicate pathways leading to PIP_2_/PA production/degradation. Dashed lines represent an induction of actin polymerization. A question mark indicates that experimental data are available only for non-plant cells. Enzymes generating PIP_2_ or PA are in green and proteins involved in phospholipid degradation or signaling attenuation are in red. In PIP_2_ and PA structural models, red and brown balls represent the oxygen and phosphorus atoms in headgroups, respectively, and carbon atoms are shown in cyan. ADF, actin-depolymerizing factor; CP, capping protein; F-actin, filamentous actin; DGK, diacylglycerol kinase; LPP, lipid phosphate phosphatases; PI4P5K, phosphatidylinositol 4-phosphate 5-kinase; PI-PLC, phosphatidylinositol-specific phospholipase C; PLD, phospholipase D; ROP, Rho of plants.

Although both PA and PIP_2_ are negatively charged (i.e., acidic) in the physiological pH range, they markedly differ in their structural and biophysical properties. PIP_2_ contains a bulky headgroup, with net charge ranging from –3 to –5 under physiological pH and an inverted conical shape that promotes positive curvature of membranes (**Figure 1**). Since total concentration of PIP_2_ in the plant plasma membrane is less than 1% (Munnik and Nielsen, 2011), PIP_2_ (together with other phosphoinositides, PPIs) is believed to function as an address label that defines membrane identity and as a landmark molecule for its protein partners, rather than having a general structural role in the lipid bilayer.

In contrast to the distinct structure of PIP_2_ that makes it very distinguishable in the membrane for its interaction partners, PA represents the simplest glycerophospholipid, consisting of a hydrophobic diacylglycerol (DAG) body and a single phosphate as the polar hydrophilic headgroup (**Figure 1**). PA is more abundant than PIP_2_ in the plant plasma membrane (usually between 5 and 10% of total phospholipids; Furt et al., 2010) and can change local properties of the lipid bilayer due to its cone-like shape, favoring negative membrane curvature (Kooijman et al., 2003; Testerink and Munnik, 2011). Interestingly, the specificity of PA interactions with its binding proteins is the result of a unique PA property called the electrostatic/hydrogen bond switch, where the negative charge of the PA headgroup is increased from –1 to –2 and stabilized upon formation of hydrogen bonds with arginine and lysine residues of effector proteins (Kooijman et al., 2007).

In addition to differences in polar headgroups, distinct membrane properties of PIP_2_ and PA may also result from different acyl compositions. In tobacco leaves, PA is predominantly made of palmitic and linoleic acid, whereas PIP_2_ contains mainly palmitic, stearic, and oleic acids (Furt et al., 2010).

## TIGHTLY REGULATED AND DISTINCT POOLS OF PIP_2_ AND PA ARE INVOLVED IN ACTIN REGULATION

### PIP_2_ PRODUCTION

Phosphoinositides biosynthesis begins with the formation of phosphatidylinositol (PI), which is produced by the condensation of cytidine-diphosphodiacylglycerol and D-*myo*-inositol in the endoplasmic reticulum (ER) (Löfke et al., 2008). The inositol ring of PI can be further phosphorylated at D-3, D-4, and D-5 position by specific evolutionarily conserved lipid kinases (Brown and Auger, 2011). The key enzyme in PIP_2_ synthesis is phosphatidylinositol 4-phosphate 5-kinase (PI4P5K). In *Arabidopsis*, 11 genes encoding PI4P5K isoforms were identified (Mueller-Roeber and Pical, 2002). These genes could be further divided into two subgroups based on their overall structure, one group containing *AtPI4P5K1–9* and the other formed by *AtPI4P5K10–11* (Ischebeck et al., 2010). PI4P5Ks have an essential role in root-hair growth, pollen development, and guard cell opening (Munnik and Nielsen, 2011). Intriguingly, a double mutant of *PI4P5K10* and *11* has increased sensitivity to actin-monomer binding drug latrunculin B, whereas overexpression of these isoforms causes aggregation of apical actin fringe in tobacco pollen tubes (Ischebeck et al., 2011), suggesting that PIP_2_ produced by this group of PIP4P5Ks is specifically involved in the regulation of actin dynamics.

In addition to PPI formation, reduction in PPI levels is also likely to regulate the actin cytoskeleton. Phosphoinositide-specific phospholipase C (PI-PLC) is an enzyme that hydrolyzes PIP_2_ into DAG and inositol trisphosphate (IP_3_), and was shown to affect actin organization in *Petunia* pollen tubes by knockdown studies (Dowd et al., 2006). Moreover, two non-related families of phosphatases are present in plant genomes: inositol polyphosphate 5-phosphatases (5PTases), that can cleave both PIP_2_ and inositol polyphosphates, and PPI phosphatases containing SAC domain that preferentially cleave membrane PPIs. Interestingly, the *fra3* mutant that has been identified as 5PTase15 implicated in controlling actin organization and secondary cell wall synthesis in fiber cells (Zhong et al., 2004). Actin disorganization was also shown in *fra7* mutant, coding for SAC-bearing PPI phosphatase (Zhong et al., 2005).

### PA PRODUCTION

In addition to ER-localized biosynthesis of PA that serves as a precursor for structural phospholipids and triacylglycerols, two distinct pathways can lead to formation of PA with signaling properties. The most studied pathway involves hydrolysis of structural phospholipids by phospholipase D (PLD), directly yielding PA. In comparison to yeast and animal genomes, the PLD family is expanded in plants with 12 genes in *Arabidopsis* and even more in other dicot and monocot genomes (Eliáš et al., 2002; Pleskot et al., 2012a). Interestingly, the PLDβ1 isoform from *Arabidopsis* and tobacco was found to interact directly with actin and is implicated in the regulation of actin polymerization (Kusner et al., 2003; Pleskot et al., 2010).

In addition to the PLD pathway, PA can be also produced by phosphorylation of DAG from the activity of diacylglycerol kinase (DGK). Intriguingly, “signaling” DAG in plant cells can be generated either from PIP_2_ via PI-PLC or from structural phospholipids via the activity of non-specific PLC (Munnik and Nielsen, 2011; Pokotylo et al., 2013), thus linking PPIs and PA signaling. The knowledge about plant DGKs is scarce and no molecular or genetic data are available that would support a role in actin regulation. However, several animal DGK isoforms have been implicated in actin regulation, and a plant DGK activity was found to be associated with F-actin in carrot cell cultures (Tan and Boss, 1992).

## MULTIFACETED ROLE OF PIP_2_ IN THE REGULATION OF ACTIN CYTOSKELETON

There are several different ways that PIP_2_ can affect actin polymerization, dynamics, and association with the membrane: through direct binding and regulation of distinct ABPs, indirectly through regulation of the activity and localization of ROP (Rho of plants) GTPases, or via recruiting scaffolding proteins to the plasma membrane (Zhang et al., 2012).

Actin-binding proteins were among the first proteins whose biological activity was shown to be regulated by PIP_2_ (reviewed in Zhang et al., 2012). There seems to be a clear distinction between inhibiting and activating properties of PIP_2_ in actin polymerization, such that all PIP_2_-sensitive G-actin-binding and actin-severing proteins are inactivated by PIP_2_, whereas for proteins acting in actin assembly or linking the filaments to the membrane, their interaction with PIP_2_ leads to increased actin polymerization and/or membrane attachment (Saarikangas et al., 2010). In contrast to the majority of PPI-binding non-cytoskeletal proteins, which have structurally well-defined PPI-binding motifs, like pleckstrin homology (PH), Phox homology (PX) or Fab-1, YGL023, Vps27, and EEA1 (FYVE) domains, most ABPs do not possess obvious structural modules, but they instead use patches of basic/aromatic amino acids, e.g., heterodimeric capping protein (CP) contains such clusters on the C-terminal parts of both subunits (Kim et al., 2007; Pleskot et al., 2012b, see also below for details).

A number of PPI-regulated ABPs have been studied in animal cells including members of ADF (actin-depolymerizing factor)/cofilin, profilin, twinfilin, CP, gelsolin, villin, α-actinin, vinculin, talin, spectrin, ERM (ezrin/radixin/moesin), and actin nucleating protein families (Saarikangas et al., 2010). In plants, four distinct ABP classes (profilin, ADF/cofilin, CP, and villin) have been described to be regulated by PIP_2_ to date (Gungabissoon et al., 1998; Braun et al., 1999; Dong et al., 2001; Xiang et al., 2007).

Profilin is a globular protein of low molecular mass, which forms a 1:1 complex with G-actin (Kovar et al., 2000). Profilin suppresses spontaneous nucleation of actin and prevents assembly at the slow-growing, pointed end of actin filaments (Staiger and Blanchoin, 2006). In contrast to non-plant counterparts, plant profilin does not catalyze nucleotide exchange on actin (reviewed in Day et al., 2011). Profilin colocalizes with PIP_2_ at the tip of growing root hairs (Braun et al., 1999). Moreover, plant profilin directly binds PIP_2_(Kovar et al., 2001) and it could be speculated that similar to its animal homologs, profilin can then dissociate from profilin-G-actin complexes releasing free G-actin (Witke, 2004). Interestingly, plant profilin also inhibits the activity of PIP_2_-degrading enzyme, PI-PLC (Kovar et al., 2000).

Proteins of the ADF/cofilin family represent conserved ABPs across eukaryotes (Hussey et al., 2002). ADF/cofilin recycles actin monomers by severing and creating new filament ends (Andrianantoandro and Pollard, 2006; Henty et al., 2011). *Zea mays* (Zm) ADF3 directly binds and is inhibited by PIP_2_. Moreover, similar to the profilin-PIP_2_ interaction, the ZmADF3 binding of PIP_2_ suppresses the activity of PI-PLC (Gungabissoon et al., 1998). Similar findings were reported for ADF1 from lily pollen (Allwood et al., 2002), suggesting that PPI regulation is a common feature of plant ADF/cofilin isoforms.

Villin belongs to the ABP protein superfamily gelsolin/villin/fragmin and is composed of six gelsolin-homology domains at its core and a villin headpiece domain at its C-terminus. *Arabidopsis* contains five *VILLIN* genes, however, genes coding for gelsolin and fragmin are not present in model plant genomes. Interestingly, actin-severing activity of ABP29, a probable splice variant of the 135-kDa villin from lily, was shown to be inhibited by PIP_2_ (Xiang et al., 2007). However, the analogous regulation of full-length plant villin remains to be demonstrated.

Capping protein is a heterodimeric protein distributed across almost all eukaryotes (Pleskot et al., 2012b); it binds to the fast growing end of actin filaments, thus inhibiting polymerization. CP bound to actin filaments also protects against disassembly (Huang et al., 2003). Similar to animal cells, it was shown that the ability of *Arabidopsis* CP to bind actin fast-growing ends is inhibited PIP_2_ *in vitro* (Huang et al., 2006). However, unlike animal and yeast CPs, the *Arabidopsis* CP homolog has been also identified as a direct target of PA both *in vitro* and *in vivo* [see below for more details; (Huang et al., 2006; Li et al., 2012a)].

Rho of plants small GTPases are a plant-specific subfamily and sole members of the Rho/Rac/Cdc42 family of Ras-related G-proteins in plants, where they serve as “master switches” involved in diverse signaling and developmental pathways. Activated ROP variants are associated with the plasma membrane, where they are thought to control cell growth by coordinating actin organization and membrane trafficking (Mucha et al., 2011). Importantly, PIP_2_ was shown to colocalize with ROP GTPases at the apical plasma membrane of tobacco pollen tube and pollen ROP physically interacts with PI4P5K activity (Kost et al., 1999; Yalovsky et al., 2008). Importantly, type II plant ROP GTPases have a polybasic motif at the C-terminal part of the protein, which is necessary for plasma membrane localization (Lavy and Yalovsky, 2006). It is therefore tempting to speculate that this polybasic motif binds PIP_2_ directly, as described for many members of the human small GTPase family (Heo et al., 2006). Furthermore, it was recently shown that PIP4P5K regulates actin dynamics in pollen tubes by counteracting Rho-GDI (Rho-guanine nucleotide dissociation inhibitor), thereby regulating the pool of membrane-localized ROP GTPases (Ischebeck et al., 2011).

## PA REGULATES PLANT ACTIN CYTOSKELETON DYNAMICS THROUGH CP

In the last decade, several studies describe changes in signaling PA levels that generate a pronounced effect on plant actin cytoskeleton organization (Lee et al., 2003; Motes et al., 2005; Huang et al., 2006; Apostolakos et al., 2008; Pleskot et al., 2010, 2012a). Given the profound effect of PA production on actin polymerization in eukaryotes, it is surprising that no ABPs regulated by PA were described in animal or yeast cells. Indeed, the PA effect on actin in animals appears to be mainly indirect, by controlling production of PIP_2_ through PI4P5Ks [(Roach et al., 2012); and see below]. In plant cells on the other hand, CP was found to be regulated by PA as well as PPIs *in vitro* (Huang et al., 2006). Furthermore, the critical role of PA in plant CP regulation was confirmed by utilizing *cp* knockdown mutants (Li et al., 2012a,b). Structural aspects of the AtCP inhibition by PA highlight a key role for the C-terminal part of CPα subunit, as demonstrated through molecular dynamics simulations (Pleskot et al., 2012b). The fact that a direct interaction between actin and PLDβ exists in plant cells (Pleskot et al., 2010) leads to the hypothesis of a positive feedback loop model for actin dynamics regulation by PLDβ and PA. Briefly, intracellular or intercellular signals cause activation of PLDβ and subsequently increase the local PA concentration. PA binds CP and prevents its binding to the fast growing end of actin filaments, thus promoting actin polymerization. Newly formed actin filaments promote PLDβ activity, leading to local enhancement of PA concentration and further enhancement of actin assembly (Pleskot et al., 2010, 2013).

## CONCLUDING REMARKS AND HYPOTHESES

During the last 20 years, multiple direct and indirect interactions between PIP_2_- and PA-centered signaling pathways and the regulation of actin dynamics have been revealed. Despite the fact that the regulation of actin dynamics is a point of convergence for many signaling pathways and exhibits complex feedback regulation (**Figure 1**), general conclusions can be drawn: The elevation of PIP_2_ and/or PA levels increases both density and complexity of the actin network and conversely the inhibition of PA/PIP_2_ production leads to actin filament disruption. Although many similarities can be found in ABP–phospholipid regulation between plant and animal cells, there is one principal difference: in plants, PIP_2_ levels are 10 times lower than PA levels (Drøbak, 1993; Zonia and Munnik, 2004). It is therefore tempting to speculate that many plant ABPs adapted to the distinct levels of PA and PIP_2_. It might be expected that additional ABPs interact with PA and/or PIP_2_ in plant cells, and this should be a topic for future exploration.

Many published reports on ABP–phospholipid regulation assume that the protein–lipid bilayer interaction is mono-specific, i.e., a single species of lipid is responsible for recruiting a given ABP to the membrane. However, work from animal and yeast cells has shown that a mono-specific reaction is the exception rather than the rule: for the majority of lipid effectors, membrane translocation probably depends both on a specific lipid but also on the surrounding lipid environment (Moravcevic et al., 2012). Indeed, several recent computational studies, albeit not on proteins involved in the regulation of the actin dynamics, show the involvement of other phospholipids for protein domains previously thought to function in a mono-specific way. Kooijman et al. (2007) experimentally described the positive effect of phosphatidylethanolamine on the PA binding by AtPDK1, AtCTR1, and Raf-1. Similar results were obtained for the binding of PPIs by PH, PX, and FYVE domains (Psachoulia and Sansom, 2008, 2009; Lumb et al., 2011). Several PA-binding proteins also have affinity for different PPIs. The binding of another signaling phospholipid could be mediated by the same domain, as in the case of AtPDK1 and p47phox PX domain, or through a completely distinct domain, for example the C1 and C2 domains of mammalian PKCε (Testerink and Munnik, 2011), but the molecular details are largely missing. Interestingly, the C1-domain, a canonical DAG-binding motif, binds more strongly to DAG embedded in the negatively charged membrane and DAG-mediated targeting of effector proteins thus seems to be also enhanced by synergistic binding to acidic phospholipids, such as PA and PIP_2_ (Colón-González and Kazanietz, 2006). From this point of view, dual regulation of plant CP by both PA and PIP_2_ (Huang et al., 2006) might represent just the tip of an iceberg.

A cooperative effect between PA and PIP_2_ in the regulation of the actin dynamics could be also indirect. Recently, Roach et al. (2012) described the ability of PA to activate PI4P5K and the authors showed the crucial importance of membrane targeting of PI4P5K by PA in the regulation of actin reorganization in animal cells. The activation of kinase activity by PA was shown for AtPI4P5K1 (Perera et al., 2005). Given the fact that several PLD isoforms are activated by PIP_2_(Li et al., 2009), one can expect that a vivid crosstalk between PA and PIP_2_ signaling to the actin cytoskeleton exists in all eukaryotic cells.

## Conflict of Interest Statement

The authors declare that the research was conducted in the absence of any commercial or financial relationships that could be construed as a potential conflict of interest.
